# Sexual and reproductive health needs and risks of very young adolescent refugees and migrants from Myanmar living in Thailand

**DOI:** 10.1186/s13031-017-0130-0

**Published:** 2017-11-14

**Authors:** Catherine Lee, Yasmina Aules, Samira Sami, Paw Kree Lar, Jennifer Schlect, Courtland Robinson

**Affiliations:** 1 Johns Hopkins Bloomberg School of Public Health, Department of International Health, 615 N. Wolfe Street, Baltimore, MD 21205 USA; 2 Adolescent Reproductive Health Network, Mae Sot, Thailand; 3 Women’s Refugee Commission, 122 East 42nd Street, New York, NY 10168 USA

**Keywords:** Thailand, Myanmar, Very young adolescents, Sexual and reproductive health, Refugees, Migrants, Conflict

## Abstract

**Background:**

The very young adolescent (VYA) population age 10–14 years is often neglected in the field of sexual and reproductive health (SRH) research due to the combined sensitivity of the topic and the young age group, resulting in little data about the SRH needs and concerns of VYA. In 2013, the Women’s Refugee Commission (WRC), Johns Hopkins University (JHU), Adolescent and Reproductive Health Network (ARHN) and Karen Youth Organization (KYO) implemented qualitative participatory research to explore the SRH needs and risks of VYA. The study was conducted in Mae Sot town and Mae La refugee camp, both in Thailand, with migrant populations and refugees, respectively.

**Methods:**

A total of 22 focus group discussions (FGD) were conducted with 176 participants. FGD were implemented with girls and boys aged 10–16, and adults in both settings. The FGD with 10–14 year olds included community mapping and photo elicitation interviews. These activities gathered information about their own perspectives, experiences and values regarding SRH, as well as SRH risks. The FGD with 15–16 year olds and adults focused on their perspectives regarding the SRH needs and risks of VYA.

**Results:**

Fourteen (64%) of FGD were conducted in Mae Sot town, and 8 (36%) were conducted in Mae La refugee camp.

Schools, youth centers and religious institutions were identified as key locations for obtaining SRH information. Schools are most promising, but access to schools is unequal between boys and girls. Parents can provide support and education to adolescents if they are supported to do so and if trust and comfort can be built between adolescents and parents around SRH.

**Conclusions:**

To a large degree, the same themes emerged from both locations, in terms of the awareness of body changes and puberty, the centrality of peer influences, and the value of education. These findings call for rigorous study of youth-directed programs and policies that meaningfully involve key influential adults identified by vulnerable young adolescents and utilize the specific places young adolescents, themselves, voice as being critical settings for obtaining information on SRH issues.

## Background

A significant gap exists in information and data regarding the experiences, perspectives and needs of very young adolescents (VYA) age 10–14 years globally and specifically following conflict. Furthermore, the sexual and reproductive health (SRH) needs and health-seeking behavior of adolescents are yet to be understood [1, 2]. The VYA population has often been neglected in the field of SRH research due to the combined sensitivity of the topic and the young age group, resulting in little data about the specific needs and concerns of VYA regarding sexuality and fertility [3, 4]. Although research on the SRH of VYA is gaining increased attention globally [5], data are still lacking regarding the needs of refugee and migrant VYA in humanitarian settings, who are at particularly high risk of adverse SRH outcomes [6].

In response to this gap in research, the Women’s Refugee Commission (WRC) and the Johns Hopkins Bloomberg School of Public Health (JHU), funded by the U.S. Centers for Disease Control and Prevention (CDC), and in partnership with Adolescent Reproductive Health Network (ARHN) and Karen Youth Organization (KYO) in Thailand, explored SRH needs and risks of VYA residing in Mae La refugee camp and migrant communities in Tak province, Thailand.

This paper focuses on describing the sexual and reproductive health needs and risks of VYA. Specific research objectives for this study included understanding the perceived SRH risks and needs of VYA, the effects of displacement on the transition from young adolescence into later adolescence and adulthood, as well as available resources and assistance for VYA in terms of community, school, family, and services.

The study sites were both in Tak Province, Thailand. Tak province is located on Thailand’s northwestern border with Myanmar; as a major transit and destination point for migrant workers, Tak is home to over 120,000 migrants, most of whom are undocumented persons living in and around Mae Sot District, a major crossing-point from Myanmar [7]. Migrant workers from Myanmar in Thailand experience various forms of exploitation, including lack of legal protections, and verbal, physical, and sexual abuse by employers and authorities [8, 9]. Children of migrant workers may live with their families or may be living in boarding houses or shelters, separated from family members by circumstances of displacement or due to parents/adult family members working in other locations in Thailand. In addition to the migrant population, as of December 2015, an estimated 106,321 people who fled Myanmar, some more than two decades ago, were living in camps on the Thailand-Myanmar border, including 38,288 in the largest camp, Mae La, to the north of Mae Sot [10].

A study conducted in 2012 assessed reproductive health on the Thailand-Myanmar border and included information on access to SRH services in both the migrant and refugee communities, as well as in internally displaced communities inside Myanmar [11]. Most relevant to adolescent SRH were the findings that, in migrant and camp settings, common barriers to accessing SRH services such as family planning counseling, supplies, and procedures included biases based on age, marital status, and security threats. In this report, organizations providing services also reported that even when services are available to unmarried persons and adolescents, the majority of their clients are married – which reflects a common perception that unmarried or young people do not need family planning supplies or counseling, or other SRH information and services. Despite some availability of adolescent reproductive health trainings and workshops, as well as a limited number of drop in centers, the report recommended increased efforts to address adolescent reproductive health needs including increasing availability of family planning services and counseling and strengthening capacity to provide services to adolescents and coordinate efforts between organizations. However, this report did not look specifically at the sexual and reproductive health risks and needs of VYA, leaving a continued gap in our understanding of the situation for this specific age population. Our study aimed to understand the SRH needs and risks of VYA in both of the humanitarian settings – migrant and camp – from the perspective of VYA, adolescents, and adults. A secondary aim was to compare the similarities and differences between responses regarding migrant and camp VYA populations’ SRH needs and risks.

## Methods

### Design and setting

In September and October of 2013, focus group discussions (FGD) and participatory methods were employed to better understand the lived realities of VYA related to the SRH-related topics of gender roles, safety, risks, community norms and expectations. The specific participatory research methods used were photo elicitation interviews and community mapping. Photo elicitation involved showing respondents several photographs staged to depict locally relevant scenarios. These were then used to elicit discussion. Community mapping involved facilitating the drawing of a physical map to identify specific places and then discuss and explore VYA interactions with these places. The framework guiding the study’s data collection and analysis was adapted from the *Global Early Adolescence Study* [12, 13]. Key domains of inquiry included: macro-level factors, community, schools, peers, family, and individual.

FGD sessions conducted with 10–12 and 13–14 year olds included community mapping and photo elicitation interviews to obtain information about their own perspectives, experiences and values as related to sexual and reproductive health, as well as risks related to SRH. FGD were also implemented with 15–16 year olds to gather their impression of SRH risks and needs during the time period from 10 to 14 years. Adolescents 15–16 years of age were included to increase information about SRH-related experiences from the time period of 10–14 years from adolescents who recently passed through this phase and could reflect back on SRH needs and risks. Finally, FGD were conducted with adults to consider the community perspectives on the SRH needs and risks of VYA. Adults included in this study were primarily parents, but teachers, health workers and camp committee members also participated.

### Participant recruitment

Two sites were included to increase the ability to compare and contrast data from individuals with similar background living in separate, but closely related and geographically nearby, settings. The migrant community around Mae Sot, Thailand and Mae La Refugee camp just north from Mae Sot and along the border with Myanmar were included. In both sites, purposive sampling was used to select participants. Potential participants were identified and recommended by local contacts from ARHN in Mae Sot and KYO in Mae La as individuals who would likely be interested in the study and could contribute unique and rich information of value to the study.

In Mae Sot, ARHN selected in-school adolescents and adults from the areas where their organization provides services, as well as from migrant schools, boarding houses, and less accessible communities out of ARHN program areas. ARHN worked with a local boarding house coordinator and contacts in the community to recruit participants from the areas outside ARHN program areas. Through these efforts, in-school and out-of-school adolescents were both included. Adults in Mae Sot were also recruited by ARHN through their existing networks. Inclusion criteria included being a migrant from Myanmar or displaced child or adult living outside of refugee camps in either Mae Sot District or Phop Phra District.

In Mae La camp, KYO conducted recruitment and selected adolescents from three different schools. Schools were selected based on geographic coverage in the camp, as well as agreement from school leaders to participate in the research. Due to security concerns of having interviewers work outside of the schools, out-of-school adolescents could not be recruited from the general camp population. KYO also recruited adults who interacted with or were responsible for 10–14 years old adolescents. Inclusion criteria included being a displaced child living in Mae La refugee camp, or an adult living in Mae La refugee camp with knowledge about the experiences of adolescents in the camp.

Local partner organization staff first approached schools and adults such as boarding house leaders to explain the project and obtain agreement to continue the research. Prior to data collection, verbal informed consent was obtained from all adults and parents or guardians of adolescents, as well as verbal assent from all adolescents. All individuals approached consented to be included in the study.

### Data collection

Four data collectors, two from ARHN and two from KYO, received a four-day training on qualitative research in Mae Sot. The data collectors were all female and ranged in age from 22 to 31 years. The training contained topics such as study background, research ethics, participant consent, facilitation skills, and note taking. Data collectors also role-played the mapping exercise and pilot tested the pictures. A follow-up training was provided a week after the first training to review procedures, as well as distribute all final data collection tools and supplies. Translators who worked independently from the two local partner organizations translated the tools into Myanmar and Karen languages.

In Mae Sot, data collection was done in Myanmar language and took place in the ARHN youth center. In Mae La camp, data collection was conducted using Karen language and took place in private, youth friendly spaces inside the camp. In both locations, the research team and local contacts felt that the ARHN Youth Center and youth friendly spaces inside the camp were suitable for data collection. In Mae Sot, transportation was arranged with the school for participating adolescents and, for those out of school, the research team arranged transportation to and from the FGD. Adults made their own arrangements for transportation to the youth center. In the camp, the youth friendly spaces were within walking distance of the schools included in the study and were familiar and acceptable to the participants. Data collectors worked in pairs, with one working as the facilitator and the other as the note taker. In both sites, all sessions were audio recorded.

For all data collection activities, the main themes focused on understanding significant influences on the behaviors and views of adolescents, how adolescents learn about the roles of men and women, what the expectations of adults are for boys and girls while comparing and contrasting similarities and differences, as well as exploring how 10–14 year olds feel about safe spaces, the health of their community for adolescents, factors that influence the SRH of adolescents, access to health services and barriers to health services.

First, the 10–14 year old adolescents undertook community mapping. They were given a flip chart paper and asked to draw a map of their community that showed important places for their age group. Then, facilitators handed out colored markers and asked participants to identify and circle the following: homes; places of learning; places of worship; places where youth socialized; and health centers and hospitals. Participants were then led through a guided discussion to talk about youth socialization, issues of safety in their communities, and access to support and information. After a break, participants were shown a series of photographs used as a starting point to have discussions about different topics. There was a total of 12 pictures which described several situations (a young girl walking alone, a young boy walking alone, a group of boys and girls walking together, a girl at the school, a boy at the school, a young girl holding a baby, a pregnant girl wearing a school uniform, hands holding, a man grabbing a woman’s arm, a dirty home, a family inside a house and a woman with a baby at the clinic). Facilitators asked what adolescents saw in the photographs and participants discussed issues such as the importance of education, relationships, body changes, differences between boys and girls in daily activities, domestic violence and also security concerns. On average, each group activity took approximately two hours.

Participatory activities with 15–16 year old adolescents followed a structure similar to the one described above. The main difference was that older adolescents were not asked to draw a map, but, rather, were shown community maps drawn by their younger peers and then encouraged to observe and discuss what they saw in the maps and reflect back on when they were 10–14 years old. Following this, participants were shown the same series of photographs described above and guided in the same discussion. Each group activity took roughly one and a half hours.

Additionally, four FGDs were conducted with adults, two per site, who were engaged to discuss and share their perspectives about the roles and relationships of young adolescents in their community. Each adult FGD took approximately 1 h.

### Analysis

An initial local language analysis [14, 15] was conducted in Mae Sot immediately following data collection. Data from both sites were combined and analyzed together by an analysis team comprised of the four data collectors, the Project Coordinator and the Assistant Project Coordinator from JHU and one translator. All data were analyzed using a process of grouping findings under common themes. Each pair of data collectors used their own local language transcripts and read through the answers to each question, identifying relevant information and key words, as well as noting important quotes. In rotation, data collectors shared information from their transcripts with the rest of the team to combine and expand on themes as they emerged. JHU facilitated the discussion with support from the translator and recorded notes. Analysis was conducted in Myanmar language with oral translation to English for note taking. Following this process, all audio files were sent for transcription and translation into English.

In-depth analysis of the English transcripts was later conducted in a two-step process using NVivo (QSR International, Version 11.1.1). In the first step, the coder conducted open coding based on a read of all transcripts. This coding was done with a line-by-line inductive coding of adolescent and adult focus groups to determine broad themes independent of the early adolescence framework described above. After the open coding, the coder and a member of the research team (Lee) developed a final codebook based on the preliminary code list developed during the open coding and further categorized the broad themes into five domains based on the framework from the *Global Early Adolescence Study* [12, 13]. These domains were: (1) *community*, such as references to community assets including sexual and reproductive health services, safety and physical environment, collective socialization, or cultural beliefs and attitudes; (2) *school* relating to any mention of access to health education or school health programs; (3) *peers* relating to youth’s descriptions of romantic relationships or behaviors and expectations; (4) *family* relating to parental involvement, family composition or support; and (5) *individual* relating to decision-making, sexual identify, health literacy or physical and mental development. In the second step, we conducted focused coding by applying the final code list to code all transcripts. Coded transcripts were then queried for findings relating to early adolescence for each of the domains, and analytical memos were created at that time.

## Results

### Participant characteristics

A total of 22 focus groups were conducted with 176 participants enrolled in the study. More than half (*n* = 14, 64%) of focus groups were conducted in Mae Sot town, and 36% (*n* = 8) were conducted in Mae La refugee camp (Table 1). There was equal participation by boys (*n* = 48, *n* = 72) and girls (n = 48, *n* = 72) in Mae Sot and Mae La, respectively. In Mae La, the groups consisted of mixed groups of boys and girls, whereas in Mae Sot the groups were divided between boys and girls. The ages of participants ranged from 10 to 16 years of age among adolescents, and 18 years and older among adults.

**Table 1:** Focus Group Discussion Characteristics

Characteristics	Female Only	Male Only	Mixed Gender
Number of participants	48	48	80
Number of focus groups	6	6	10
Age Group			
10–12 years old	2	2	2
13–14 years old	2	2	2
15–16 years old	2	2	2
Adults	0	0	4
Location			
Mae Sot	6	6	2
Mae La	0	0	8

### Emergent themes

Table 2 lists the emergent themes for each of the five early adolescence domains described by adolescents and adults regarding the sexual and reproductive health needs and risks of very young adolescents. The specific themes per early adolescence domain are described below. The emergent themes and concepts align with the domains from the early adolescence framework explained above that served as the overarching framework for the study.

**Table 2:** Focus Group Emergent Themes for Early Adolescence

Early Adolescence Domain	Emergent Theme	Early Adolescence Concept
Community	Neighborhood resources	*Community assets* where VYA seek services such as food, livelihood, and gender-based violence
	Community health	
	Community expectations	*Cultural beliefs and attitudes* of what is expected or appropriate for different age groups
	Community norms	
	Gender roles	
	Socialization	*Collective socialization* of how adolescents learn to think or behave in certain ways dictated by societal beliefs, values, attitudes
	Safety and security	*Safety and physical environment* of risks (perceived, feared, experienced) from harm or violence for VYA
School	Education	*Opportunities and expectations* to achieve formal education
	Learning	*Connections* with community programs, religious teachings and mentors on SRH
Peers	Peer support	*Behaviors and expectations* related to peer support
		*Norms and values* for peer intimate and non-intimate relationships
Family	Household dynamics	*Gender norms and values* of members within a household
	Household duties	*Expectations and support* for household roles and decision making of VYA
	Parental involvement	
Individual	VYA attitudes and norms	*Aspirations and expectations* of VYA in their context
	Individual aspirations	
	VYA needs	
	Body awareness	*Puberty and cognitive maturation* of VYA from childhood to adolescence
	Physical and mental health	

### Community

#### Community assets related to SRH

Places of worship were noted within all FGDs of this age group, as an enjoyable place to spend time with their peers, receive religious teachings and seek guidance on moral and personal issues. Other neighborhood resources identified by these groups included the police office, health facility, schools and several governmental and non-governmental organizations. Both male and female groups described their parents, elders and community leaders as individuals they would approach for advice about difficult questions including violence and transition from childhood to adolescence. 15–16 year olds agreed with this impression.

#### Community-level cultural beliefs and attitudes regarding premarital sexual relationships

On several occasions, adults stated that the attitudes and behaviors of current adolescents in their communities contrast greatly from their attitudes when they, themselves, were young. Adult participants explained that adolescents aged 13 to 17 are pursuing sexual relations and perceive the characters of adolescents as being ‘damaged’ following displacement from Myanmar. Although adult participants do not agree with the change in cultural norms related to sexual relations, VYA in the community were described as having sexual intercourse before marriage and using abortion to end pregnancy.

Lack of acceptance for sexual relations among adolescents under the age of 18 was shared among adults in Mae Sot and Mae La in addition to lack of acceptance for sexual relations for unmarried couples of any age. In addition, several adults felt that adolescents under the age of 15 should not be educated about sexual health until they are older. Adults also remarked that adolescents under the age of 18 of both sexes are allowed to spend time together but not live together because it puts them at risk for pregnancy. Adolescents aged 15 to 16 described that in the community they often see couples younger than themselves, holding hands and displaying affection to each other in public, whereas younger adolescents felt that romantic relationships begin at the age of 14.

#### Collective socialization

Media such as television, movies and Internet were seen as important contributors towards the attitudes and behaviors of children and young adolescents. According to adults, adolescents observe negative behaviors in movies and model those in their romantic relationships with peers. In contrast, VYA attributed their beliefs and behaviors primarily to their parents, teachers and family members. Older adolescents (aged 15–16) credited socialization during early adolescents equally to media and parents.

“We are influenced by parents, brother, sister and friends.”—Mae Sot, Girls, Aged 10 – 12

“Parents and teachers are the ones who mainly influence children.”—Mae Sot, Boys, Aged 10 – 12

Adults acknowledge their position as an important role model for influencing the adolescents’ transition from childhood to adulthood. Similarly, they agreed that young adolescents often mimic the actions of their parents so parents do have the ability to guide adolescents to make positive life decisions.

“The children start to change at the age of 8-9 years old. They will watch what you are doing and saying, and they will copy it after that. Parents should teach them well and show them the right way during this age.”—Mae La, Adults

#### Safety and physical environment

Children 10–14 in both communities reported spending the majority of their time outside of the house. Morning to afternoon hours are spent in the school and afterschool hours are spent at places of worship or play. When asked about areas that are unsafe, adolescents 10–14, as well as those 15–16 felt that the greatest risks during early adolescents was for being trafficked or raped when walking alone in the community. A clear finding shared by adolescent boys and girls of all ages was that adolescents, particularly girls, are at great risk of being trafficked if they walk in the community alone.

“Girls will be abused and raped. Therefore, it is more dangerous for girls than boys. It is also dangerous if girls walk away from the main road because people who take children live in that area.”—Mae Sot, Girls, Aged 13 – 14

Additionally, there were several discussions among male adolescents that they feel unsafe due to threats of arrests from immigration officers and police. Female and male 13–14 year olds attributed the fear of being arrested to the lack of legal documentation among displaced children and their families.

“It is not safe even at home because immigration police come to our homes.”—Mae Sot, Boys, Aged 13 – 14

### School

#### Opportunities and expectations

Access to formal education was not seen as universal for 10–14 year olds in either community. Young girls, in particular, were seen as facing the greatest challenges to attending school. Often families require the eldest girl child to stay at home during the day to care for the youngest siblings, conduct household chores, or to work. Also, some adolescents were unable to attend school because of distance, uniform costs or school fees. Lastly, the need to obtain an education was commonly discussed as an important factor in determining the quality of their future. Even at a young age, girls aged 10 to 12 voice a desire to be educated and finish schooling.

“We want to finish schooling and get a complete education.”—Mae Sot, Girls, Aged 10 – 12

“If children do not go to school, then there will be consequences for their future. If you do not have any education, then you have to do labor intensive work and will be treated badly by the work owner and rich men.”— Mae Sot, Boys, Aged 13 - 14

#### Connections

Places of formal and informal education provided very young adolescents with additional resources when seeking health information. Often times, very young adolescents referred to schools, library and youth centers as sources of information about health. Additionally, the health clinic was often a venue sought by very young adolescents for learning about SRH issues. Adolescents aged 13–16 described the clinic as a place to gain knowledge on the transition from childhood to adolescence including the topic of pregnancy prevention.

### Peers

#### Behaviors and expectations

Adults and older adolescents noted childhood as a period when young adolescents spend most of their time making friendships and spending time with their peers either at school or in recreational spaces. Moreover, both of the older age groups felt that peers are the most influential for the behaviors of VYA. This perception was consistent with other views held by the older age groups that peers should be educated about positive norms by their own peers.

“The children love to have friends and when they are playing with their friends they even forget about their parents. The youth have to teach themselves. The people who give the training should also be the youth. Youth who are good and become leaders will also be the same generation as them and should persuade them.”—Mae La, Adults

While one group of girls aged 10 to 12 in Mae Sot mentioned there is no safe place designated for adolescents in the community to play, other groups in Mae Sot and Mae La described meeting peers at youth centers and sporting fields.

#### Peer norms and values for peer intimate and non-intimate relationships

All adolescent age groups discussed the effect of pregnancy on their relationships with peers. Without probing, adolescents shared on multiple occasions that young girls would be stigmatized or ignored by their peers if they become pregnant at a young age.

“A girl who is about to deliver will receive good care from her family and friends. But her friends will stop friendship with her.”—Mae Sot, Boys, Aged 10 – 12

### Family

#### Family-level gender norms and values

The differences in the roles of girls and boys, and mothers and fathers were clearly noted by all age groups. In both communities, girls have different caretaking responsibilities for younger siblings in comparison to boys. The female child is also expected to support the mother in household chores that include cleaning whereas the boys are expected to carry heavy items such as water. Similarly among parents, the father is acknowledged as having the most power in the household and holds the responsibility of ensuring family needs are met. In contrast, the role of the mother is to care for their children and house.

“The father is the head of the family and has the most power in the family. He will take care of the whole family.”—Mae Sot, Girls, Aged 10 – 12

“In our community, it is the girl’s responsibility. She should know automatically to do the household things and provide for the family. Carrying small water tank, throwing away the garbage and lifting heavy weights are better for men. Housekeeping and scrubbing floors are better for women.”—Mae Sot, Boys, Aged 13 – 14

“Young girls are not free, they are not happy, they feel troubled and they are not allowed to go wherever they want to go. Her brother has the chance to live happily with the family. She does not have the same chances as her siblings because she has to look after her brothers or sisters. She does not have a chance to go to school.”-- Mae Sot, Girls, Aged 13 – 14

#### Expectations and support

Adults and adolescents indicated that parents would prefer for their children to aspire to higher educational degrees and professions. This concept was coupled with adolescents’ desire to attend school being restricted by a family’s economic situation.

“Family and relatives hope their children become a teacher, a doctor and an engineer…Education is the key for their ambitions. When children suffer destitution, they cannot further their education and they mostly drop out of school. The effects of dropping out of school will cause children’s to lie and steal”-- Mae Sot, Boys, Aged 10 – 12

When adolescents were asked about situations where a young girl would become pregnant, some felt that their parents would become ashamed of them but the majority of groups felt that parents would accept the pregnancy and support their child.

“A young girl got pregnant and has to live shamefully with her family. Even though her parents don’t want to accept for this to happen, they couldn’t do anything since it’s already happened. So, they have to accept it with sadness.”- Mae La, Mixed group, Aged 13 - 14

Similarly, nearly all adolescent groups identified their parents as people from whom they would seek information about the transition of their body from childhood to adolescence. Adolescents reported that they would seek advice from their parents on general health matters, but they would go to a health clinic to learn more about how to prevent pregnancy. Adolescents who were married were perceived as being able to discuss family planning with their parents.

### Individual

#### Puberty and cognitive maturation

For adolescents aged 10 to 12, most age groups perceived them to be ‘children’ that are still unaware of the physical changes in their body. Changes in adolescent’s behaviors and attitudes are perceived to be more evident between the ages of 15 to 17. Although the opinion is not exclusive to adult groups, the most evident transition from childhood to adolescence is between 10 to 14 year olds and 15 to 17 year olds. Adults further described age 15 as the moment when children experience change in hormones, and exhibit independence in decision-making with more interest in intimate relationships with the opposite sex.

“For the age of 15-17, they would want to get married and don’t really listen to their parents anymore. They become more independent and stubborn.”—Mae La, Mixed group, Adults

## Discussion

The results indicate that these experiences are more similar than different, when comparing the refugee context of Mae La camp, with the informal migrant setting of Mae Sot. Despite the relative stability of Tak province in Thailand, this research identifies that VYA in both of the displaced settings continue to experience a significant amount of stress and strain resulting from issues of safety and security, both of which can impact SRH risks for exploitation and sexual assault and abuse. In addition to family separation resulting from migratory labor, VYA are acutely aware of risks of trafficking /abduction and rape that they face.

Additionally, adolescents and adults report that many young people become sexually active late within this period of early adolescents, or shortly thereafter. However, very little SRH information appears to be openly available to this age group [13], and parents do not feel comfortable discussing this topic until adolescents approach 17 or 18 years of age. Shame, disapproval and abortion were mentioned frequently in the context of pregnancy, which suggest high risks of unsafe abortion for this young population.

Despite significant risks faced by this young group, there are strong community assets that can be leveraged to improve the availability and delivery of SRH information and supports. Community assets, frequently destabilized during conflict and protracted displacement, appear to be well established and supportive for VYA in both Mae La and Mae Sot. Adolescents are found to seek out places of worship for support, guidance, information and peer interaction. Schools, youth centers, and health facilities are similarly viewed as positive sources of information and support. Adolescents in this study reported trusting the health center in their community, as an information source. Since VYA typically have few reasons to come for health care before facing pregnancy, SRH complications, or other infirmity, this is an incredible asset in this context. Interactions between these community structures and adolescents in their early years offer a critical opportunity for sharing information on health or well-being, and connecting VYA to SRH programs. Since VYA themselves identified specific community locations to which they would turn for information, it is important for the individuals (e.g. teachers, religious leaders, and parents) to be equipped with relevant SRH information when such support is sought. It is equally important to measure actual utilization of services at the community locations mentioned by the respondents and rigorously measure intervention effectiveness in these settings given that most of the data supporting interventions is from high income countries and among populations not experiencing migration [16].

In these settings, adolescents view formal education as critical to achieving future goals and aspirations. However, schools were unequally accessed by boys and girls. School dropout is closely linked to a number of negative health and social outcomes. When VYA do not have access to school they lack access to peer educators and leaders who can act as positive role models and support systems to gain knowledge about SRH. Low attendance and school drop out will additionally limit opportunities to access health information on SRH that has been mainstreamed within the school curriculum.

In both locations where this research was carried out, parents maintain significant influence in the lives of adolescents, during these early years. Younger adolescents see them as a support system when they need help, particularly if they experience violence. Parents, however, acknowledge their role in shaping their child’s decisions, but they do not feel their children value or seek their guidance. Therefore, SRH programs could seek to strengthen communication and trust building between adolescents and parents while also providing parents with the support and information they need to guide their children on SRH. Given the existing evidence base for adolescent SRH programs delivered through the locations mentioned by respondents in this study (schools, youth centers, health facilities) [17] an important consideration is that evidence is still needed for program impacts for VYA and implementation of programs involving VYA and adolescents in the development of youth-directed programs. Capitalizing on the existing peer and adult social networks of VYA could lead to more sustained impact of SRH interventions that utilize community-, clinic-, or youth center-based approaches, particularly in times of displacement.

### Limitations

Focus group discussions may have limited the depth of information collected when compared to an alternative of conducting in-depth interviews. However, the study team decided on focus groups as the method for data collection in order more closely compare convergent and divergent opinions among participants. The selection of the migrant community site (Mae Sot) and the refugee camp (Mae Le) was not random but purposive to take advantage of sites where local partner organizations (ARHN and KYO) worked. While both sites have significant populations of refugees and/or migrants from Myanmar with characteristics common in other migrant communities and refugee camps in this area, results may not be generalizable to other locations. Interviewing of adolescents by age groups (10–12, 13–14, and 15–16) was consistent between Mae Sot and Mae La, though in Mae La, all the FGDs with the different adolescent age groups (*n* = 6) combined boys and girls while in Mae Sot, two FGDs per age group comprised boys only or girls only, while two FGDs per age group combined boys and girls. It is possible that different group dynamics were captured in these two sites making direct comparisons more difficult. In addition, the mixed groups of boys and girls may have caused changes in responses from either sex. However, the local partner organization in Mae La refugee camp had previous experience conducting mixed group discussions for similar topics and, in agreement with school and community leaders helping to organize, felt that these FGD could be conducted with both boys and girls together and we did not see any differences in the data or receive any feedback that the mixed group FGD were problematic for the participants. Also, while initial processing of the interview results was done as a group process, utilizing data collectors reviewing Myanmar language manuscripts and discussing findings in Myanmar language and English, the data analysis in NVivo was done with English language translations of the Myanmar language interview transcripts. Thus, some nuance and meaning of the original comments could have been lost or misinterpreted in the English-language analysis. Finally, analysis did not include looking at the similarities or differences in responses from in-school and out-of-school participants.

## Conclusion

One intention of this study was in comparing results from interviews in a refugee camp (Mae La) with interviews in a nearby migrant community (Mae Sot), to see how these different contexts and displacement/migration experiences shaped VYA perspectives on SRH needs and risks. What we found was that, to a large degree, the same themes emerged from both locations, in terms of the awareness of body changes and puberty, the centrality of peer influences, and the value of education. Perhaps this is not surprising if we consider that both types of migration from Myanmar have been protracted, motivated by decades of conflict, human rights abuse and hardship. Equally important, conditions for both refugees and migrants in Thailand – while far preferable to Myanmar – have offered both meaningful sustenance, through migrant labor or refugee relief, while also perpetuating experiences of marginality and risk, leading us to define both types of movement as forms of “crisis migration” [18]. Indeed, compared to the refugee camp, boys and girls in Mae Sot expressed more concerns about their physical safety, with girls fearing rape and abuse and boys fearing arrests from immigration officers and police. These two populations, living in such close proximity to one another, and sharing so much of a common history of displacement and a current reality of marginality call for youth-directed programs supported by both the humanitarian and development communities to study the effectiveness of SRH programs and policies for vulnerable young adolescents in migration. These new approaches could involve programs and policies that meaningfully involve key influential adults identified by vulnerable young adolescents and utilize the specific places young adolescents, themselves, voice as being critical settings for obtaining information on SRH issues. Coupled with the approaches for this type of vulnerable youth population is the need for systematic and rigorous study of the interventions in order to increase the evidence-base for SRH programs for adolescents in low resource settings, and specifically in migration.

## Abbreviations

ARHN: Adolescent reproductive health network
CCT: Community consultation team
FGD: Focus group discussion
IRB: Institutional Review Board
JHU: Johns Hopkins University
KYO: Karen Youth Organization
SRH: Sexual and Reproductive Health
VYA: Very young adolescents
WRC: Women’s Refugee Commission
